# First-in-Human Implantation of Gutter-Free Design Stent-Graft in *in situ* Fenestration TEVAR for Aortic Arch Pathology

**DOI:** 10.3389/fcvm.2022.911689

**Published:** 2022-06-30

**Authors:** Xin Li, Chang Shu, Lunchang Wang, Quanming Li, Kun Fang, Mingyao Luo, Weichang Zhang, Yang Zhou, Haiyang Zhou

**Affiliations:** ^1^Department of Vascular Surgery, The Second Xiangya Hospital of Central South University, Changsha, China; ^2^Institute of Vascular Diseases, Central South University, Changsha, China; ^3^Vascular Surgery Department, National Center for Cardiovascular Disease, Fuwai Hospital, Chinese Academy of Medical Sciences and Peking Union Medical College, Beijing, China; ^4^Department of Anesthesiology, The Second Xiangya Hospital of Central South University, Changsha, China

**Keywords:** TEVAR, fenestration, gutter-free, endograft, aortic arch pathologies

## Abstract

**Purpose:**

To report the technology and preliminary result of gutter-free design stent-grafted in *in situ* fenestration thoracic endovascular aortic repair (TEVAR).

**Description:**

The gutter-free stent-graft has a nickel-titanium self-expanding skeleton, double polytetrafluoroethylene coating, and an outer-skirt fabric structure (named C-skirt endograft). The outer skirt fabric prevents endoleak from the gutter around the stent graft fenestration. Further, the skirt structure right under the fenestration in the aortic stent graft can function as a fixation of the side-branch artery endograft. These designs have the following advantages, such as: 1) prevention of endoleak; and 2) fixation tightly between the branch and aorta endograft pieces.

**Evaluation:**

A patient who was diagnosed with an aortic arch aneurysm, combined with localized dissection, has successfully implanted the aortic stent graft and C-skirt endograft for the left subclavian artery. The 6-month follow-up result of the C-skirt in situ fenestration TEVAR is satisfactory without obvious endoleak.

**Conclusions:**

The new gutter-free C-skirt stent graft is being safely and effectively used for aortic arch TEVAR. Long-term evaluation of safety, effectivity, and durability needs to be proven by future multi-center studies.

## Technology

Surgical arch replacement is the gold standard for aortic arch pathologies, whereas the in situ fenestration technique combined with TEVAR, is a less invasive approach with lower in-hospital mortality and complication rate. However, technique difficulties, demand for various devices, and endoleak are still challenges for the in situ fenestration TEVAR. Here, we designed a dedicated device system and technique special for in situ fenestration TEVAR.

The novel gutter-free stent-graft device for supra-aortic branches named the C-skirt (Lifetech Scientific, Shenzhen, China), has the advantage to overcome the defects of currently used branch stents or stent-grafts. And we also developed a modified aortic stent graft to favor the puncture maneuver. Furthermore, we designed a new puncture needle that facilitates membrane puncture. The previous study has confirmed the features of gutter-free branch stent-graft and puncture needle system *in vitro* model and *in vivo* experimental studies in pigs (1). The C-skirt system is currently undergoing its initial safety and feasibility evaluation by a multi-center, single-arm, and pre-clinical trial (clinicaltrials.gov ID: NCT05126446). We now report the technology and early follow-up results of this new gutter-free stent-graft implantation in *in situ* fenestration TEVAR for aortic arch pathology.

## Technique

### Patient Selection (First in Man Case)

Patients in this study should have met the indications for endovascular treatment of aortic dissection or aneurysm, according to the guideline of the European Association for Cardio-Thoracic Surgery (EACTS) and the European Society for Vascular Surgery (ESVS) (2). The ongoing study is designed for patients who are surgically high-risk with aortic pathologies involving the aortic arch, in which TEVAR alone does not fit owing to the insufficient landing zone. This prospective, multi-center, and single-arm evaluation is approved by the ethics committee of the Second Xiangya Hospital, Central South University (#LTP86-01). The requirements for inclusion criteria include: 1) Age between 18–80 years old; 2) Diagnosis of aortic dissection and reconstruction of the left subclavian artery; 3) The main branches and branches of the aortic arch at the lesion site have sufficient anatomical basis: The proximal diameter range of aortic graft: 21–46 mm; The length of the aortic anchor zone is ≥10 mm; The length of the anchor zone of the branching vessel is ≥15 mm; The diameter range of the distal anchoring zone of the branching vessel is 5–18 mm; and 4) The patient has adequate surgical access vessels.

### Device Description

**Figure 1:**
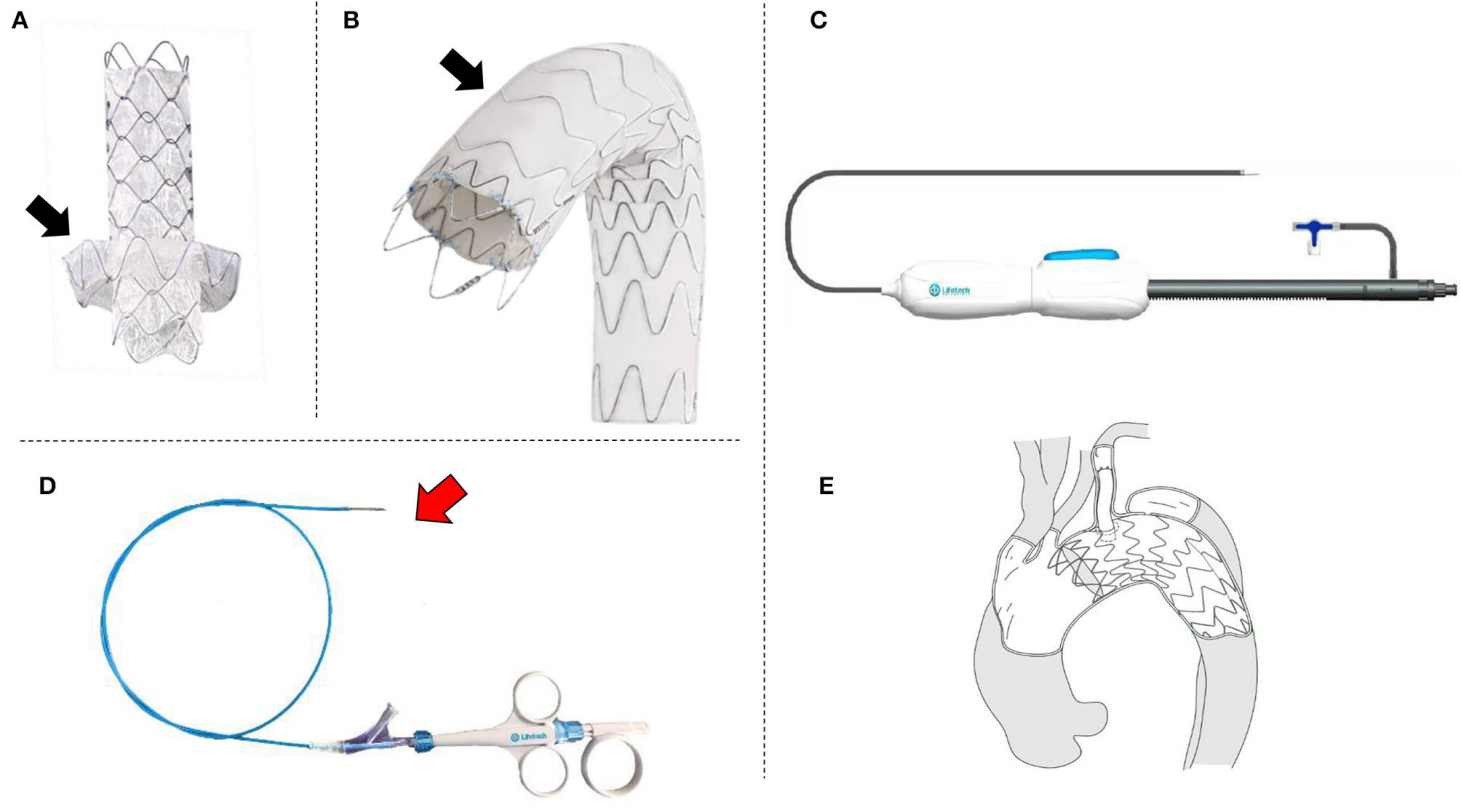
The photos of and sketch of the novel device system. **(A)** Branch stent-graft with “skirt-like” structure to prevent endoleak (black arrow); **(B)** Aortic stent-graft special designed for *in situ* fenestration, the black arrow shows the area for fenestration; **(C)** Photo of a real product of the delivery system; **(D)** The puncture needle for the *in situ* fenestration, the red arrow shows the needle; **(E)** The sketch of the system which is after the *in situ* fenestration TEVAR by using the novel stent-grafts and puncture needle system.

The C-skirt branch stent-graft is consisting of an inner stent and an “outer skirt” to form an integrated structure. The skeletal structure is a nickel-titanium self-expanding stent. The double-layer polytetrafluoroethylene membrane is mounted in the metal stent. There is a skirt-shaped structure in out layer of the C-skirt stent graft which fits the gutter to prevent endoleak (Figure 1A). There are radiopaque markers at the “skirt” structure and both ends of the stent. Also, we changed the structure of the aortic stent graft by increasing the metal wave cell area. This artificially increases the convenience of puncture of the membrane (Figure 1B). This new aortic stent graft is named Ankura Pro also made by Lifetech Scientific, Shenzhen, China. The handle of the C-skirt is divided into a fixed handle and a movable handle, a stent release locking component is assembled into the movable handle (Figure 1C). The delivery system uses an 8-Fr to 12-Fr sheath with a.035-inch guidewire. To facilitate *in situ* fenestration, we developed a puncture catheter system. It may be used in case of favorable anatomy situations and use combined with out-sheath (Figure 1D). The sketch of the deployed C-skirt *in situ* fenestration TEVAR is shown in Figure 1E.

## Clinical experience

A 60-year-old female patient is diagnosed with localized aortic dissection aneurysm without back or chest pain. She had uncontrolled hypertension, but no other comorbidities are found. Her laboratory results are normal.

**Figure 2:**
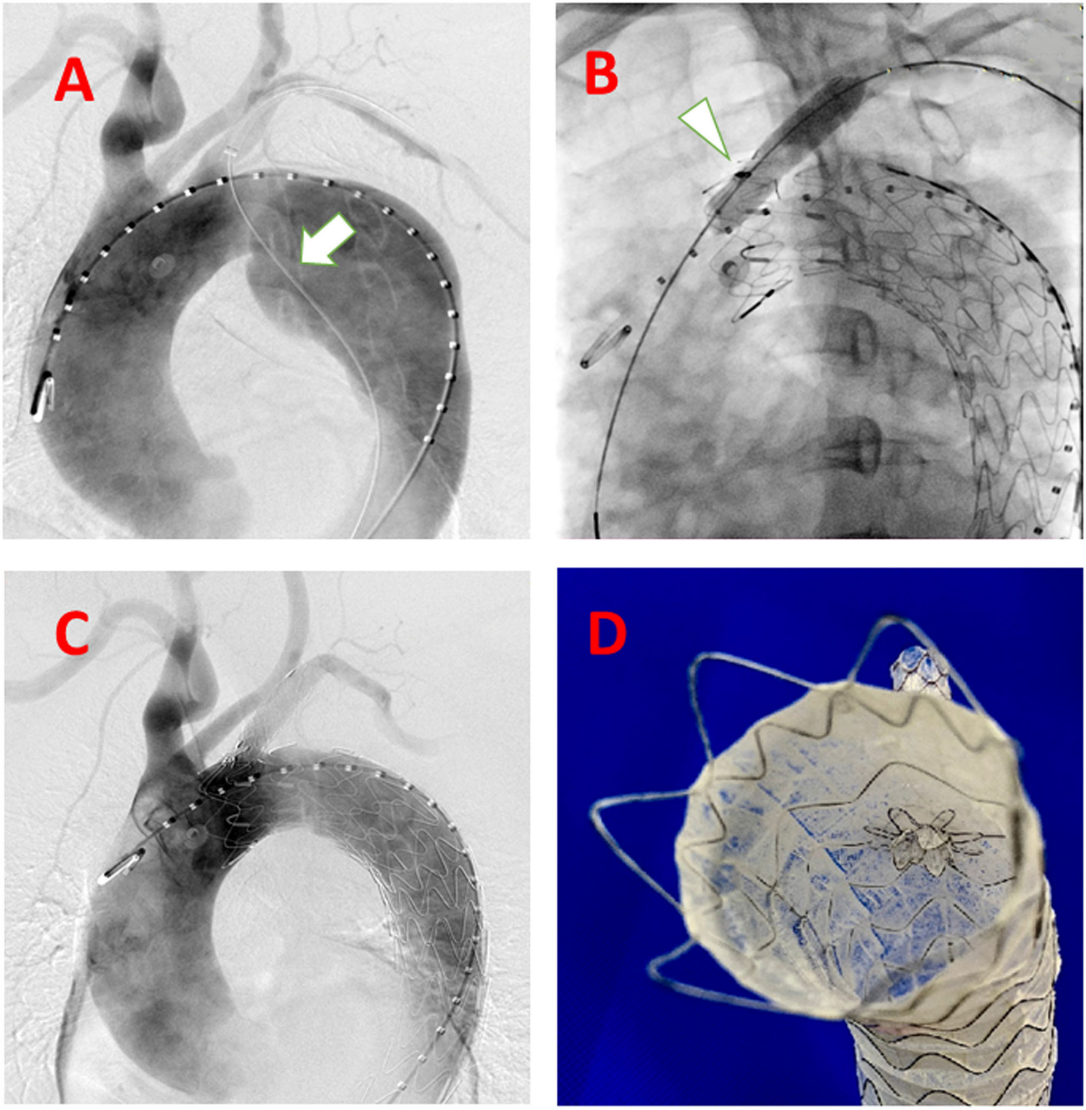
Steps of the system accomplished delivered in the human case. **(A)** guidewire (white arrow) from LSA to the descending aorta to the femoral artery as a strengthen wire, which makes puncture angle from LSA to aortic stent-graft more friendly; **(B)** After balloon expansion (white arrow) of the deployed C-skirt stent-graft; **(C)** DSA after the whole procedure accomplished; **(D)** Real view of the C-skirt stent-graft in the aortic stent graft.

**Figure 3:**
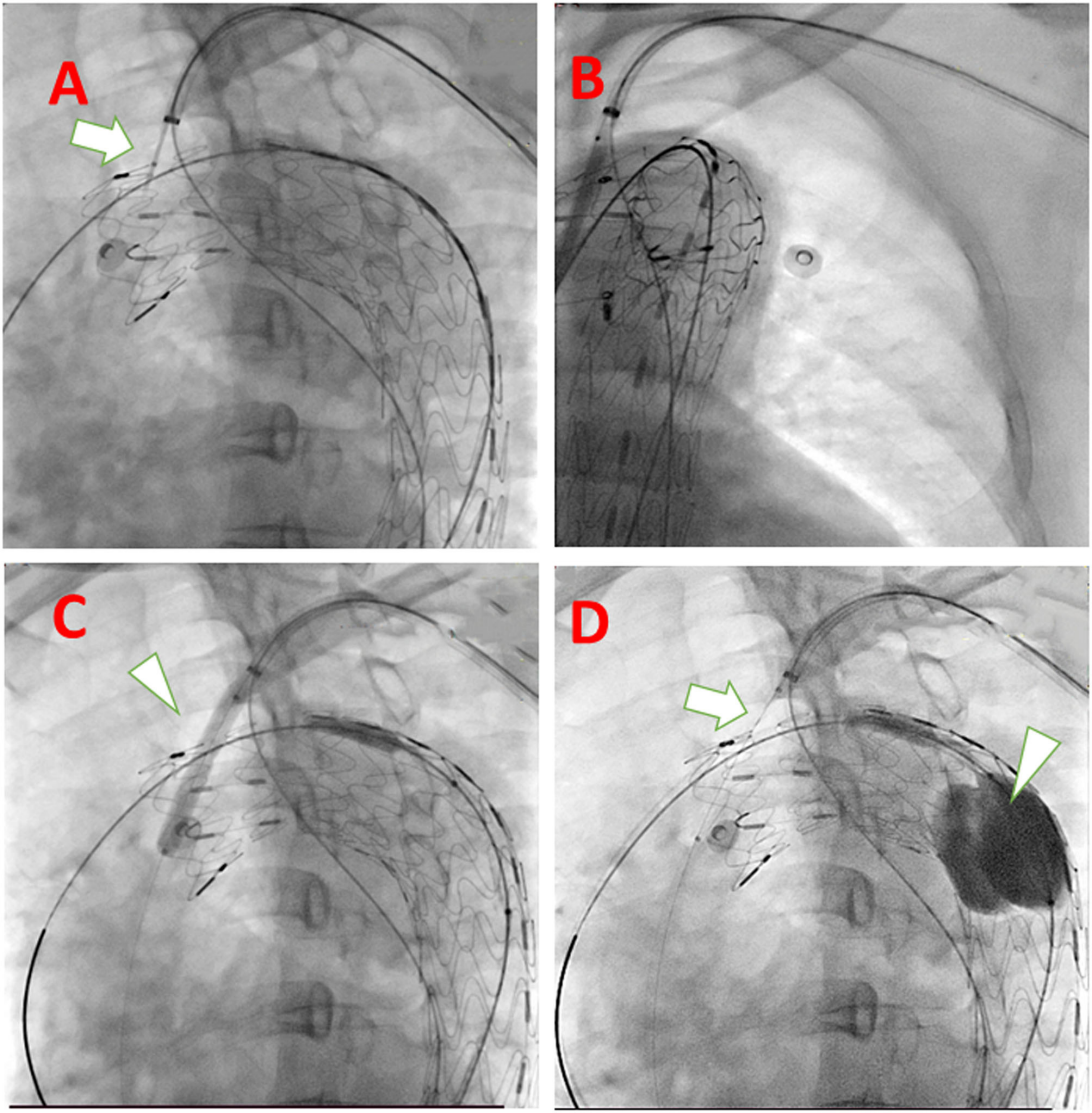
Technical spots mentioned in the system deployment procedure. **(A)** Puncture needle with descending strengthen wire in the same long sheath when puncturing (white arrow); **(B)** rotate the tube and make sure the guidewire and expanding balloon into the fenestration is in the lumen of the aortic stent graft; **(C)** Using larger diameter balloon (white arrowhead) to expand the fenestration; **(D)** Insert the delivery system of C-skirt stent graft (white arrow) and to prevent migration of aortic stent-graft, a Tri-lobe balloon (white arrowhead) is expanded if necessary.

After general anesthesia, the right femoral artery and left brachial artery were exposed surgically. A guidewire in a 10 Fr sheath reaching in proximal LSA is inserted from the left brachial artery to descending aorta to the femoral artery as a strengthen wire which makes puncture angle from LSA to aortic stent-graft more friendly (Figure 2A). After deploying the C-skirt in the LSA, the radiopaque marker in the skirt structure is just below the orifice of the LSA. A balloon is inflated in the C-skirt to make the after-dilation (Figure 2B). The “skirt” is functional in preventing endoleak and also making the C-skirt stent-graft more stable in both the aortic stent graft and LSA proximal site (Figures 2C,D). There are some technical spots we need to mention as below. After an angiogram from a pigtail catheter in ascending aorta from the right femoral artery, a 32-24-200 mm (proximal diameter-distal diameter- length) Ankura Pro aortic stent-graft is deployed in Zone 1, and the membrane-proximal border is right distal to the orifice of the left carotid artery. The puncture catheter has been sent to the proximal LSA location through another guidewire in the 10 Fr sheath (Figure 3A). When finishing the puncture through the membrane, a 0.018 guidewire has been sent to ascending aorta through the fenestration in the puncture needle. After changing the X-ray tube angle to make sure the guidewire is through the fenestration into the aortic stent-graft lumen (Figure 3B), we gradually increased the balloon diameter from 3 mm−8 mm and expanded the fenestration area (Figure 3C). Because the resistance is relatively strong when inserting a balloon and C-skirt stent-graft (10–50 mm, diameter-length), a Tri-lobe balloon (W.L. Gore & Associates, Inc. 1505 N, Fourth Street, Flagstaff, Arizona, USA) is inflated in distal aortic stent-graft for providing counterforce to avoid migration (Figure 3D).

## Comment

The endovascular treatment of aortic dissection or aneurysms involving the arch is always challenging. Compared to open surgery or hybrid operation, the endovascular treatment is with fewer perioperative complications (3). In treating these aortic arch lesions, physicians prefer to use several assisted techniques including chimney stents (4, 5), *in situ* (6, 7) or *in vitro* fenestration (8, 9), branched stent-graft (10) according to individual experience and patients' anatomy characteristics. Theoretically, the branched stent graft is the most ideal prosthesis for arch reconstruction purposes, but the shortage of this pattern is that it needs customizing according to a different configuration of the arch. The incidence of stroke of branched graft is 5.5 % to 11 % in the previous literature (11). The *in situ* fenestration technique requires surgeons to modify the structure of the original stent graft *in vivo*. In that, the puncture device needs refinement to be more efficient. The puncture catheter we use in the case is a specially designed system with the access of 0.018 guidewires. It is convenient to exchange the small diameter balloon to expand the original puncture hole in the membrane after the successful puncture.

**Figure 4:**
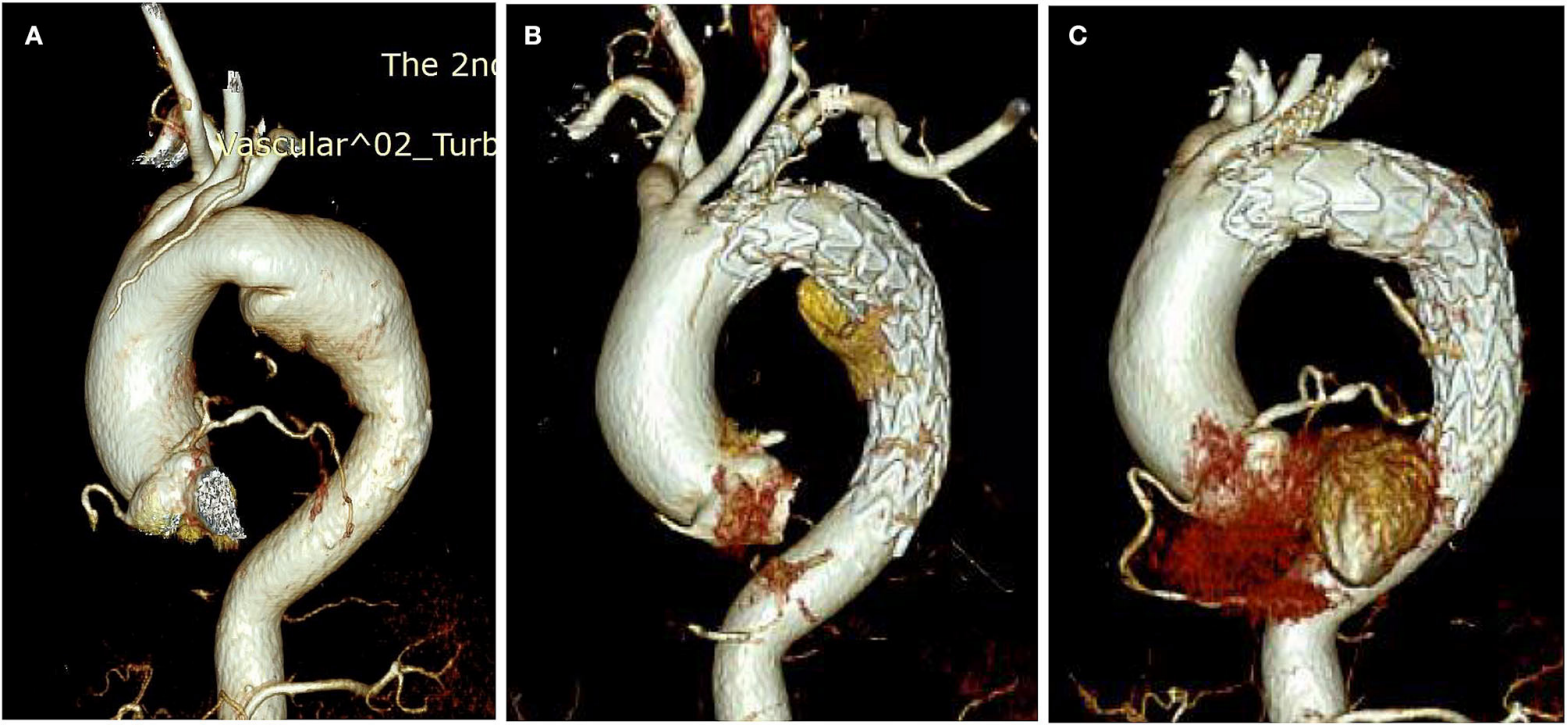
The CTA of the aorta before and after the TEVAR. **(A)** Before TEVAR; **(B)** 1-week CTA check after TEVAR; **(C)** 6 months CTA check after TEVAR.

Of the branch stent graft, there is still no specially designed stent dedicated to the *in situ* fenestration technique. Currently, we use some iliac-femoral stents and carotid stents for substitution, all of which are off-label use. Therefore, we design this C-skirt stent-graft unique for keeping branch artery patent, as well as preventing endoleak by out-skirt structure. Unlike another gutter-free stent-graft special for chimney (Longuette stent-graft) also designed by us, the skirt structure is very short and is fixed in the inner surface of the aortic stent-graft. This design makes the C-skirt prevent endoleak from fenestration gutter and get more stable fixation simultaneously. Some details need to be noted in the practice of applying this system. In our experience in this patient, only one vessel was revascularized, and theoretically, multiple branch stents would increase the probability of endoleak. For this case, there is still a small amount of endoleak afterward. We noticed that this may come from the latent persistent ductus arteriosus of this lady. We get 6 months computed tomography angiography (CTA) follow-up shows barely any endoleak anymore compared to 1-week CTA, which means the endoleak prevention function of C-skirt is not only instant after TEVAR but also in the later phase (Figure 4). The skirt-like structure as a physical “barrier” or “obstacle” in the gutter can provide permanent endoleak flow decreasing function until the endoleak diminish at last.

In conclusion, the *in situ* f-TEVAR using the novel C-skirt stent-graft is safe and effective in treating an aortic arch lesion in the short term. We shall update our data by the ongoing clinical trial.

## Data Availability Statement

The raw data supporting the conclusions of this article will be made available by the authors, without undue reservation.

## Ethics Statement

The studies involving human participants were reviewed and approved by the Ethics Committee of the Second Xiangya Hospital, Central South University (#LTP86-01). The patients/participants provided their written informed consent to participate in this study. Written informed consent was obtained from the individual(s) for the publication of any potentially identifiable images or data included in this article.

## Author Contributions

CS is conceiving and original creating. XL, LW, and QL designing and writing. KF, ML, WZ, YZ, and HZ collecting and analyse data. All authors contributed to the article and approved the submitted version.

## Funding

This study was supported by National Natural Science Foundation of China (81870345 and 81900423) and Hunan Provincial Natural Science Foundation (2020JJ2054 and 2020JJ5836). Lifetech Scientific Co. supported this study. The authors had full control of the design of the study, methods used, outcome parameters, and analysis of data and production of the written report. All authors declare freedom of investigation for this work.

## Conflict of Interest

The authors declare that the research was conducted in the absence of any commercial or financial relationships that could be construed as a potential conflict of interest.

## Publisher's Note

All claims expressed in this article are solely those of the authors and do not necessarily represent those of their affiliated organizations, or those of the publisher, the editors and the reviewers. Any product that may be evaluated in this article, or claim that may be made by its manufacturer, is not guaranteed or endorsed by the publisher.
